# Genesis of the hydrothermal gold system in the kibaran metallogenic province (D.R.Congo): A review for the Twangiza-Namoya gold belt

**DOI:** 10.1016/j.heliyon.2024.e33222

**Published:** 2024-06-18

**Authors:** Rub'son N'nahano Heritier, Huan Li, Mohammed Abdalla Elsharif Ibrahim, Claude Nambaje, Moise Luemba

**Affiliations:** a Key Laboratory of Metallogenic Prediction of Nonferrous Metals and Geological Environment Monitoring, Ministry of Education, School of Geosciences and Info–Physics, Central South University, Changsha, 410083, China; b Centre for Earth Sciences, Indian Institute of Science, Bangalore, 560012, India; c Trinity Metals Ltd, Rwanda; d School of Mining and Geology, College of Sciences and Technology, The University of Rwanda, Rwanda; e School of Geosciences, China University of Petroleum, Changjiang West Road, 66, Huangdao District, Qingdao, Shandong, 266580, China; f Faculty of Oil, Gas and New Energies, University of Kinshasa, Kinshasa, Democratic Republic of the Congo

**Keywords:** Kibara orogenic belt, Fluid inclusion, Hydrothermal gold deposits, Pan-african orogeny, Sulfur isotopes, Twangiza namoya gold belt

## Abstract

The Twangiza-Namoya Gold Deposit within the Kibaran Belt of the Democratic Republic of the Congo represents a crucial manifestation of the hydrothermal gold system. This review investigates its intricate origin and the subsequent metallogenic evolution that has shaped its present-day characteristics and offers a systematic categorization based on its deposition processes and geotectonic settings. The findings reveal that the gold deposits are predominantly derived from sedimentary fluid sources. Within this vast metallogenic province, two stages of gold deposition have been constrained: (a) the early-stage formation related to the accretion of Rodinia assembly with subduction-collisional event where diagenesis cemented the syngenetic pyrite carbonaceous sediments and (b) the later stage deposition related to the continent-collisional event during the last stage of Rodinia supercontinent amalgamation. Previous isotopic investigations, with a particular emphasis on pyrite sulfur isotopes on both host rocks and vein-bearing sulfides, have been instrumental in tracing the origins of gold-bearing fluids in the study region. The isotopic variance in the four deposits: Twangiza (−5.2 % to +3 %, avg. −0.3 %), Kamituga (−0.6 % to −0.9.1 %, avg. −5%), Lugushwa (+3.0 % to −18.4 %), and Namoya, on the southernmost end, has a vast range but with much heavier isotope compositions, ranging between +1.3 % and to +22.6 %, with an average of +12.2 %. The data predominantly points to the sedimentary origins of ore fluids in the Twangiza-Namoya Gold belt, highlighting the pivotal role of sedimentary processes in shaping the metallogenic landscape of the region. The fluids inclusions depicted the deposits to be formed from H_2_O-Nacl-H_2_O with abundant CH_4_ and N_2_ ore-forming fluid, moderate temperature (350–500 °C), and low salinity. The overall results confirm the genetic style of the Twangiza-Namoya Gold Belt to be an orogenic gold-style deposit that was emplaced during the early Neoproterozoic era in low greenschist facies terrain.

## 1 Introduction

A profound review and exploration into the origin and metallogenic evolution of the hydrothermal gold system in the Kibaran belt is significant for deciphering the system of orogenic gold. The Kibaran belt is a Mesoproterozoic orogenic belt with an intercontinental signature and a huge metallogenic province. It covers nations of the Democratic Republic of the Congo (DRC) (great Kivu region); Rwanda; Burundi; Northeastern Tanzania and the Southwestern part of Uganda [[1], [2], [3]]. The orogenic gold deposit situated within the Twangiza-Namoya belt of the Democratic Republic of the Congo exemplified a crucial segment of Central Africa's vast geological treasure trove. This zone forms a significant part of the Kibaran Belt, a Mesoproterozoic orogenic formation, which displays intercontinental characteristics and stretches across territories [[1], [2], [3]]. The belt has been the focal point of the discovery of various precious and rare metals like Nb–Ta–Sn–W and Au, which are inferred to be associated with late-stage magmatic activities during the Rodinia supercontinent's amalgamation circa 1 billion years ago [[4], [5], [6], [7], [8]]. Chronological research endeavors within this geographical context have predominantly zoomed in on the intricate nature of Nb–Ta–Sn–W metallogenic belts, deciphering their genesis, ore fluid pathways, enrichment modalities, and deposition mechanisms [7], [[9], [10], [11], [12], [13], [14], [15], [16]]. These studies have considerably amplified our comprehension of these elements in the Democratic Republic of the Congo's geological history. Paradoxically, the vast gold deposits dotted within this region have scarcely been the primary focus of scholarly explorations [3,6,17,18]. Three-fold lithostratigraphic groupings comprise the Kibaran metallogenic province (KMP).

a) The basal conglomerate and dark laminated pelitic sedimentary rocks with intercalations of siltstones, sandstones, mature quartzites, and tuffs make up the lower group (1200–1500 m). There are intrusive are noticed.
b) Minor basaltic and dacitic volcanic rocks make up the middle group (1000–2600 m).
c) The juvenile clastic layers with a basal conglomerate, ferruginous quartzite, siltstone, and shale make up the top group (>1500 m).

Within the KMP, four structurally recognizable episodes of deformation are identified [[19], [20], [21]].

a) D1 structure is an S1 schistosity parallel to the bedding, with minor isoclinal D1 folds and thrusts connected to the granite's synkinematic emplacement.
b) D2 Structure: An S2 axial planar cleavage and D2 folding are caused by NW-SE trendings. The primary structural characteristics of the Kibaran belt were caused by the Kibaran deformation, which reached its peak at this point.
c) D3 structure: at the acute angle created by renewed upright folding, N–S axes cut D2 folds; d) Structure D4: Alkaline and carbonatitic intrusions are impacted by shear folding and cataclastic deformation.

With the possible exception of migmatites, kibaran metasediments are of relatively low grade. Around granitic intrusions, high-grade metamorphism (amphibolites facies, high temperature/low-pressure type, with andalusite and staurolite in the metapelites) emerges. Furthermore [22], identified four distinct granite varieties (G1, G2, G3, and G4) in the Kibaran Metallogenic Province (KMP).

a) Syn-orogenic dates of 1370 ± 25 Ma for G1 and 1310 ± 25 Ma for G2 are given. While G2 granites lack porphyritic peraluminous orthogneiss, G1 granites are porphyritic gneissose two-micas or biotite with low Sr ratios.
b) Post-orogenic G3 and G4 are dated to around 1094 ± 50 Ma and 976 ± 10 Ma, respectively, and are accompanied by an abundance of pegmatites. G4, also known as tin granites, is accompanied by a large number of pegmatites and veins bearing Au, Sn, W, Nb–Ta, Be, Cu, Pb, tourmaline, and U mineralization. In contrast, G3, an alkaline biotite granite, is thought to have originated from a rift.

According to Ref. [21], these G4 are sub-alkaline, leucocratic, equigranular, aplitic, or pegmatitic, and extremely peraluminous. Three mineralization types associated with the KMP's development and across the Karagwe-Ankole Belt (KAB) have been identified [19,23].

a) mineralization includes Ni–Cu–Co–Fe–V–Ti–Au-PGM and sulfides found in the 1275 Ma mafic layered intrusions of Kabanga-Musongati;
b) The following mineralization is connected to the tin granites (G4): Pegmatites (Ta–Li–Be–U–Sn–Nb), quartz veins (Sn–W–Au–U–Bi-Pyrite-Siderite), and quartz veins, breccias, and auri-ferric silicification zones which all include native Au-pyrites and arsenopyrites.
c) Alkaline complexes and carbonatites are associated with the mineralization of pyrochlores and REE deposits.

The Lufilian-Zambezi orogeny (950-800 Ma) indicates the end of the Kibaran orogeny. Subsequent occurrences include the Pan-African resetting of mineral ages (600–500 Ma), the creation of the Itombwe supergroup (900–500 Ma), and the intrusion of alkaline complexes (700–500 Ma) and carbonatites (ca. 500 Ma) [21,24,25].

**Fig. 1:**
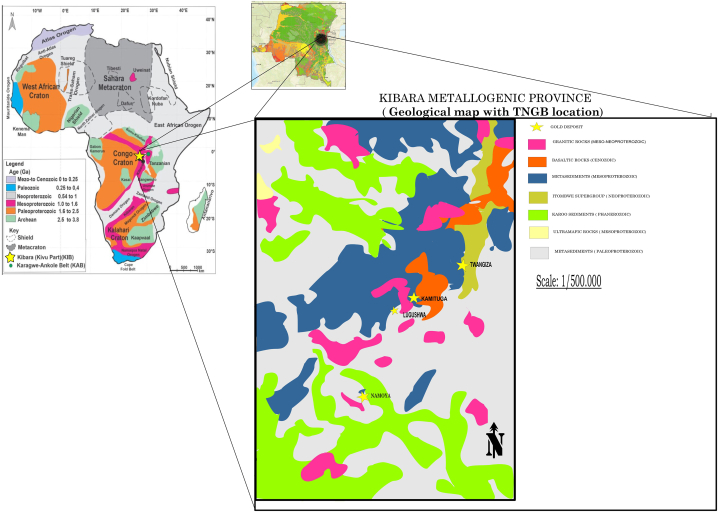
Geological map of the Kibaran Metallogenic Province showing the TNGB location in DRC (modified from Ref. [64]).

Twangiza-Namoya Gold Belt (TNGB) is located in the Kivu region and administrative provinces of South Kivu and Maniema. The TNGB comprises the Twangiza-Kamituga-Lugushwa-Namoya gold major deposits (Fig. 1). Twangiza and Kamituga are 139 km distance. Kamituga and Lugushwa are distanced from each other by 50 km. Namoya is located around 210 km southwest of the Twangiza deposit.

[21] systematically deciphered four types of inclusions within the Twangiza gold deposits. H_2_O-Nacl–CO_2_–salt fluid; H_2_O–NaCl–CO_2_ fluid; H_2_O–NaCl-Salt fluid and H_2_O–NaCl fluid. He evidenced that the H_2_O–NaCl–CO_2_ ± salt fluid system consisted of a drop of pressure and an opening of a lithostatic regime that are related to the primary Au and As ore formation. In contrast, the fluid system of H_2_O–NaCl ± salt has consisted of a drop in temperature and a break in the hydrostatic regime. He correlated the later effect to the secondary Au–As and Sb ore formation mechanism. He concluded that this changeability of temperature and salinity showed by inclusions inferred that fluid ore sources are taken root from admixture of meteoric-magmatic and meteoric waters. He proposed a genetic model for the Twangiza gold deposit to be the result of the magmatic degassing hydrothermal fluids that connected with partial meteoric and/or connate fluids with some combination of basinal waters during the gold deposition.

A Pan-African age of 530 Ma has been assigned to the gold mineralization [2,26,27]; an early Neoproterozoic age [28] associated with the main Kibaran deformation event. As noted by Refs. [1,2], the geodynamics of the Kibaran models have been proposed as both an intracratonic protracted extensional setting with anorogenic bimodal magmatism around 1375 Ma and two far-field compressional events at 1 Ga and 550 Ma, as well as a subduction-collision setting with convergence and compression at 1375 Ma coupled with asthenospheric upwelling and delimination eventually evolving into a continent-continent collision at 1Ga [8,29,30].

According to Ref. [31], the fluids responsible for the mineralization of gold in the TNGB originated from the devolatisation of the greenstones that were deeply buried in Archean to early Proterozoic formation formations. These greenstones were channeled by significant channels to the upper levels of the crust. Additionally, a paragenetic model of the Twangiza deposit was put forth by Ref. [32], indicating that albitites entered the Kibara belt at a time of approximately 750 Ma, postdating the G4 granites that were deposited at approximately 975 Ma.

Sedimentation continued throughout the Neoproterozoic, resulting in the glacial diamictites at a time approximately 800 Ma. Folds with a N–S direction were formed by the folding of abilities and sedimentary rocks during the Pan-African orogeny. Consequently, gold-bearing fluid from the lower crust's devolatization of the lower crust reached structural transition because of the strong heat flux and local structural compressional events. In addition, upon examining quartz veins carrying gold and linked with the tourmaline in the Kamituga and Lugushwa deposits [27,33].

[33] deduced that the hydrothermal gold system in the TNGB derived significantly from the fluid supplied by metasedimentary host rocks. A proposition that enough heat was available during the early Pan-African period to generate fluid activity in the central region of the TNGB that was regionally important was proposed. Afterward, the secondary inflow fluids that postdating tourmaline-bearing quartz veins were noticed. Sadly, though, they were unable to determine the source of the gold due to the subsequent observations.

In further investigation of the fluid's genesis in the TNGB [34], concluded that the hydrothermal veins in the TNGB originated at the temperature of 390–480 °C and a pressure of 1.2–2.1 Kbar, corresponding to 4.7–8.2 Km below the surface. Secondary fluid inclusions were commonly seen in significant abundances in gold mineralization. He suggested that the subsequent fluid influx, rather than the primary vein formation was responsible for the gold mineralization.

In the TNGB deposits, sulfur isotopes on pyrites in host rocks and veins carrying sulfides related to gold ore were investigated by Ref. [35]. He concluded that the host rocks containing pyrites had a main sedimentary to evaporitic source of sulfur, based on isotopic data. Both sedimentary host rocks and vein samples contained the native gold in the TNGB. However, In Lugushwa deposit result showed that the sulfur and potential fluid carrying gold were taken from an igneous source, while other deposits showed that sulfur was taken from the sedimentary host rocks. He inferred that gold and sulfur were mobilized in the sedimentary host rocks and precipitated in the TNGB hydrothermal system. Furthermore, in Namoya gold fields, veins formations at the Imoga gold deposit were investigated by Ref. [36].

Three successive generations of veins were constrained at the Imoga gold deposit. The first-generation vein formed before the folding and the two subsequent generation veins occur after folding [36]. They found that in the second and very infrequently in the third generations, gold appears as free gold or is combined with pyrite. The early Neoproterozoic (980 Ma) compressional deformation event, linked to the merger of the Rodinia supercontinent, is thought to have caused the gold mineralization at Imoga. This interpretation is based on the paragenesis, structural features, and the relationship between veining and metamorphic minerals.

Despite the KMP's evident mineralogical abundance, a coherent, regionally attuned gold metallogenic model for the region remains conspicuously absent. This lacuna arises chiefly from a deficiency in systematic studies dedicated to understanding the geochemical and structural frameworks that influence gold deposits. Thus, a profound discernment of the genesis and characteristics of gold ore fluids, especially in the backdrop of orogenic activities, continues to be a challenge. Addressing this salient knowledge deficit, our paper embarks on an exhaustive scrutiny of existing literature, intent on devising a nascent conceptual genetic gold metallogenic model pertinent to the Twangiza-Namoya gold belt in the Democratic Republic of the Congo.

By melding and critically evaluating the existence corpus of knowledge, we aim to construct a resilient foundation for future investigative and explorative undertakings in this geologically ample region. This review is envisaged not only to plug the existing academic lacunae but also to augment our understanding of the Democratic Republic of the Congo's geological and economic prospects in housing invaluable gold reserves. By fostering such in-depth scientific inquiries and exploration, we anticipate triggering more sustainable harnessing of the Democratic Republic of the Congo's vast geological assets.

## 2 Geological background

### 2.1 Twangiza-Namoya gold belt

#### 2.1.1 Twangiza gold fields

**Fig. 2:**
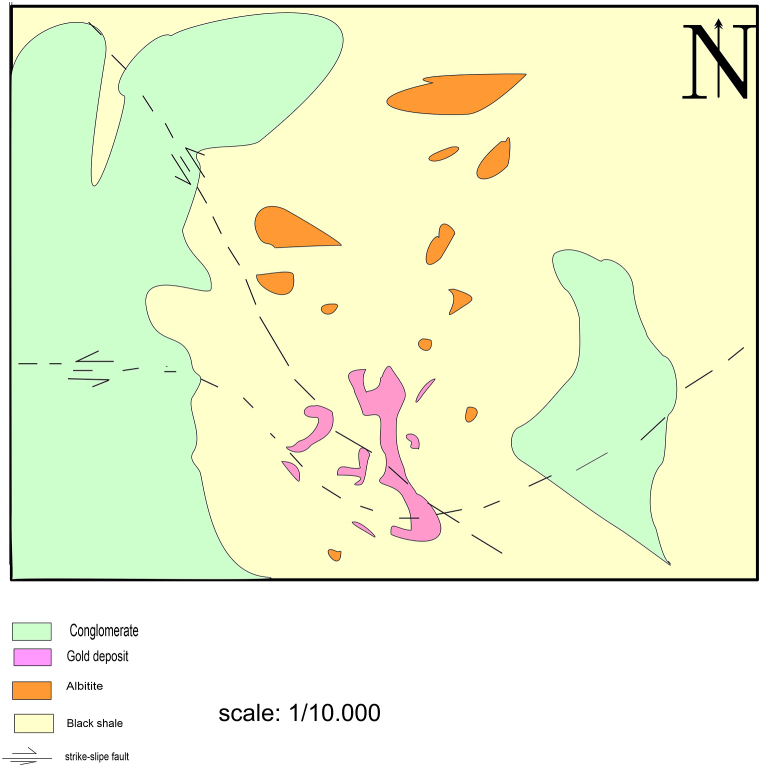
Geological map of Twangiza gold fields (modified from Ref. [21]). (For interpretation of the references to colour in this figure legend, the reader is referred to the Web version of this article.)

A series of fine-grained, folded, weakly metamorphosed clastic strata host the Twangiza deposit. Thin sills ranging in thickness from 2 to 50 m penetrate the clastic deposits. Mafic and feldspar porphyry are the two types of sill-like shape intrusions that are distinguished [37]. From bottom to top, the sedimentary succession is composed of black shales, mudstones, siltstones, sandstones, and conglomerates. Furthermore, the predominant lithology of Twangiza is composed of mudstones with a high carbonaceous content [31]. Within the major Twangiza ore deposit, sulfides can be found in the host rocks'veins and as disseminations. The two primary sulfides are arsenopyrites and pyrites. Mineralization has been linked to porphyry sills in certain areas of the ore body (Fig. 2).

Quartz, albite, chlorite, sericite, plagioclase, epidote, actinolite, biotite, hematite, goethite, and limonite are the minerals that define the Twangiza deposit. Furthermore, the deposit contains common accessory minerals constituents such as calcite, ankerite, apatite, graphite, magnesite, rutile, talc, and Kaolinite, as well as tourmaline and zircon [21]. According to the quoted author, Neoproterozoic granite magmatism was thought to be a sign of hydrothermal activity in the Twangiza deposit. He used ^39^Ar/^40^Ar ages of 529 ± 5 Ma, 436 ± 8 Ma, and Rb/Sr ages of 703 ± 14 Ma to date the age of granitic rocks.

#### 2.1.2 Kamituga gold fields

**Fig. 3:**
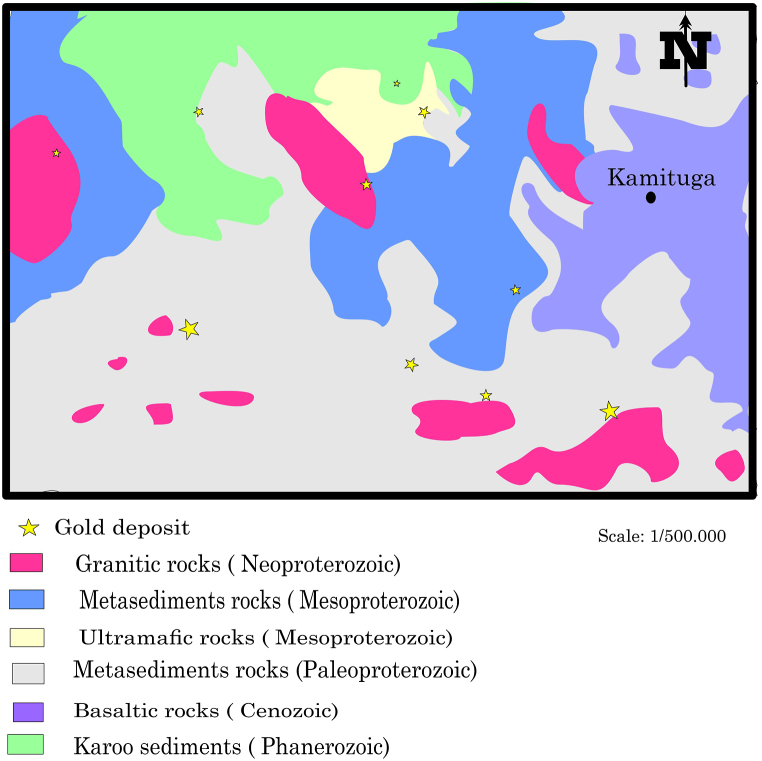
Simiplified geological map of Kamituga gold fields (modified from Ref. [64]). (For interpretation of the references to colour in this figure legend, the reader is referred to the Web version of this article.)

The thicks pelitic schist succession that intercalates with semi-pelitic biotite-muscovite schists dominates Kamituga's geological formations. According to Ref. [3], the primary intrusive bodies are the meta-diorites and G4 granites, which are associated with pegmatites and greisen. Shists are changing into phyllites or quartzites in the zone where the temperature is higher than that of the greenschist facies [31] (Fig. 3). The key lithological units that contain the primary gold mineralization are schists and quartz veins. Schists, quartzites, quartz-phyllites, amphibolites, pegmatites, and tourmaline make up the orebody. The quartz-phyllites, sericite, Kaolin, and graphite occasionally have an iron oxide banding pattern. High-grade quartz veins have been associated with gold mineralization, according to Ref. [38]. It is believed that the wall rock's notable mineralization originates from quartz veins. The veins contain modest sphalerite and other sulfides, galena, and stibnite in addition to pyrites and arsenopyrites.

#### 2.1.3 Lugushwa gold fields

**Fig. 4:**
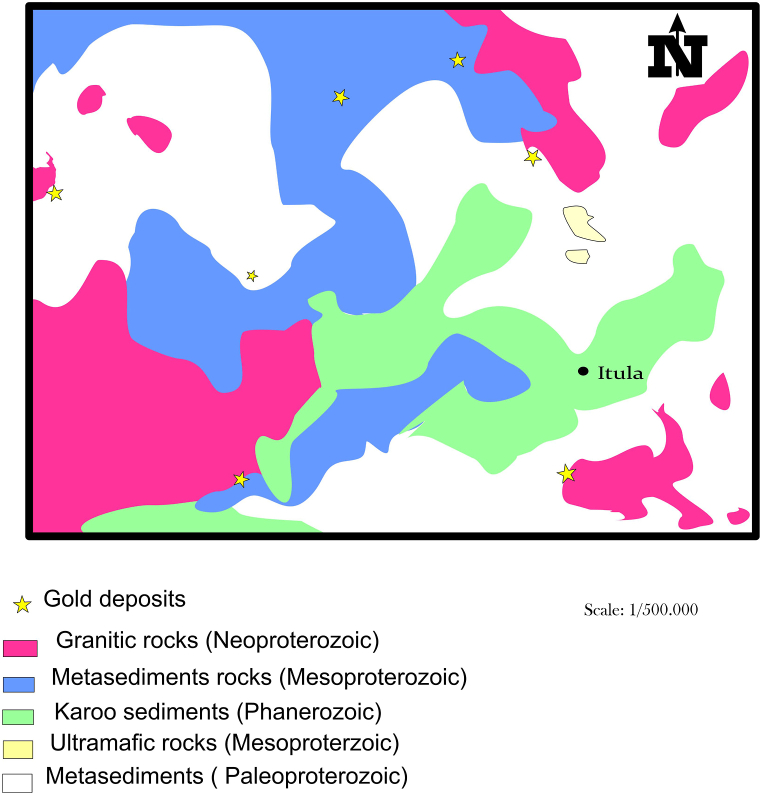
Geological map of Lugushwa gold fields (modified from Ref. [64]). (For interpretation of the references to colour in this figure legend, the reader is referred to the Web version of this article.)

The Lugushwa terrane is a facies of low greenschist. The primary geological formations with prominent foliations are the metasedimentary schists. Quartzites and sandstones with schists (mica and chlorite schists) interbedded are the lithologies [39]. Tourmaline and garnet are found in the heavily altered schist strata. Sn mineralization is related to granites and pegmatites [3]. Quartz veins and stockworks are linked to gold mineralization, distributed arsenopyrite, tourmaline, biotite, and chlorite changes are particularly noticeable in folds and hinges at the boundaries between interbedded metapelites and metasiltstones [31]. Zones with intercalated metabasites, quartzites, sandstones, and sulfides (arsenopyrites) containing schists are where gold is found throughout the deposit. While homogeneous metapelites are less mineralized or have no mineralization, schistose metapelites with quarzitic interlayers are correlated with gold grades [35] (Fig. 4).

It should be noted that while scattered gold mineralization in host rock schists is occasionally observed, arsenopyrite is not usually linked to it. A small number of sulfides, carbonates, tourmalines, micas and in rare instances, barite and cassiterite can be found in mineralized quartz veins [3,31,34,35].

#### 2.1.4 Namoya gold fields

**Fig. 5:**
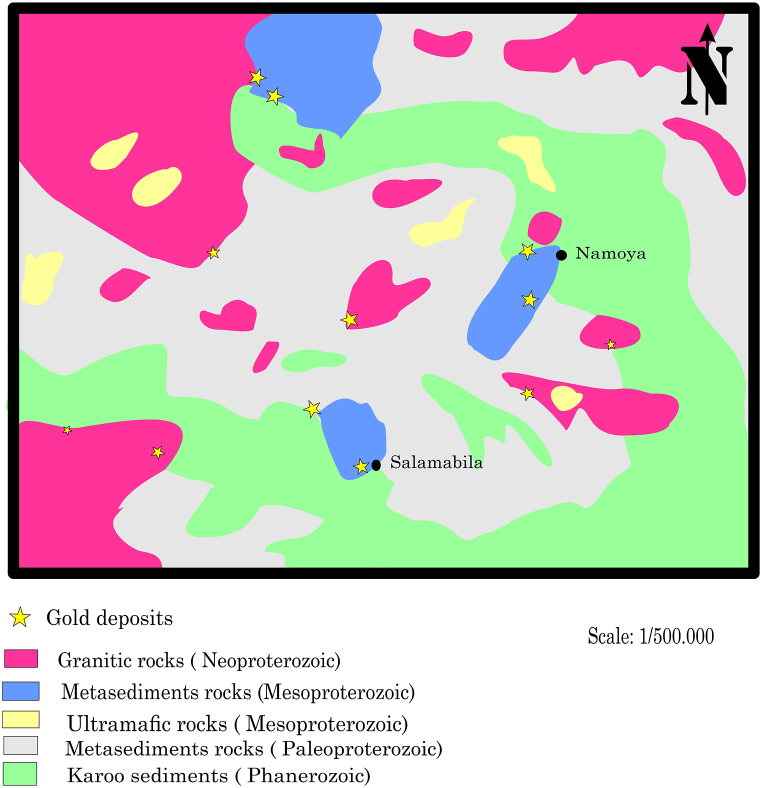
Simiplified geological map of Namoya gold fields (modified from Ref. [64]). (For interpretation of the references to colour in this figure legend, the reader is referred to the Web version of this article.)

Schists, metapelites, conglomerates, gneiss, amphibolite, pyroxenites, and feldspar-rich quartzites make up the deposit. Compared to Lugushwa and Kamituga deposits; the metamorphism grades in Namoya 6 are for low to mid-greenschist facies which is a little higher [36]. The types of metasedimentary rocks in Namoya are graphitic and silicified schists. the nature of intrusive rocks are mostly dolerite and quartz-porphyry (Fig. 5). According to Refs. [31,34], alteration assemblage is defined as silicification and sulfidation of host rocks. Metaschists encroached upon by sills and dykes hosting quartz veins and stockworks. The deposit's mineralization appears to be structurally restricted over a 2.5 km NW-SE trending corridor that is dominated by stockworks zones and irregular quartz veins hosted by sericite schists [35].

Along shear zones, the ore body is contained in E-W quartz veins. structures created during brittle-ductile shearing are thought to have undergone mineralization [39]. Arsenic, tin, barium, lead, and copper are linked with the high value of gold in quartz veins. Pyrites and arsenopyrites are the main minerals linked to mineralization; tourmaline and calcite are secondary minerals found in close proximity to quartz veins [31,35,39].

## 3 Genesis and metallogenic evolution of TNGB

In order to give a first metallogenic model and to decipher a shared understanding of the manner of gold deposition in the KMP, this scientific study attempts to synthesize earlier completed scientific studies in TNGB by adding a wide understanding description of each deposit and reassessing the gold ore system in this promising metallogenic province in Central Africa. The data used to support the interpretations of this work came from the following sources [3,34,35]: and a published PhD thesis by Ref. [21]. We therefore advise the audience to go therein for more details regarding sample descriptions, analysis techniques, and further supplementary materials.

### 3.1 Fluid inclusions (FIs) studies

**Fig. 6:**
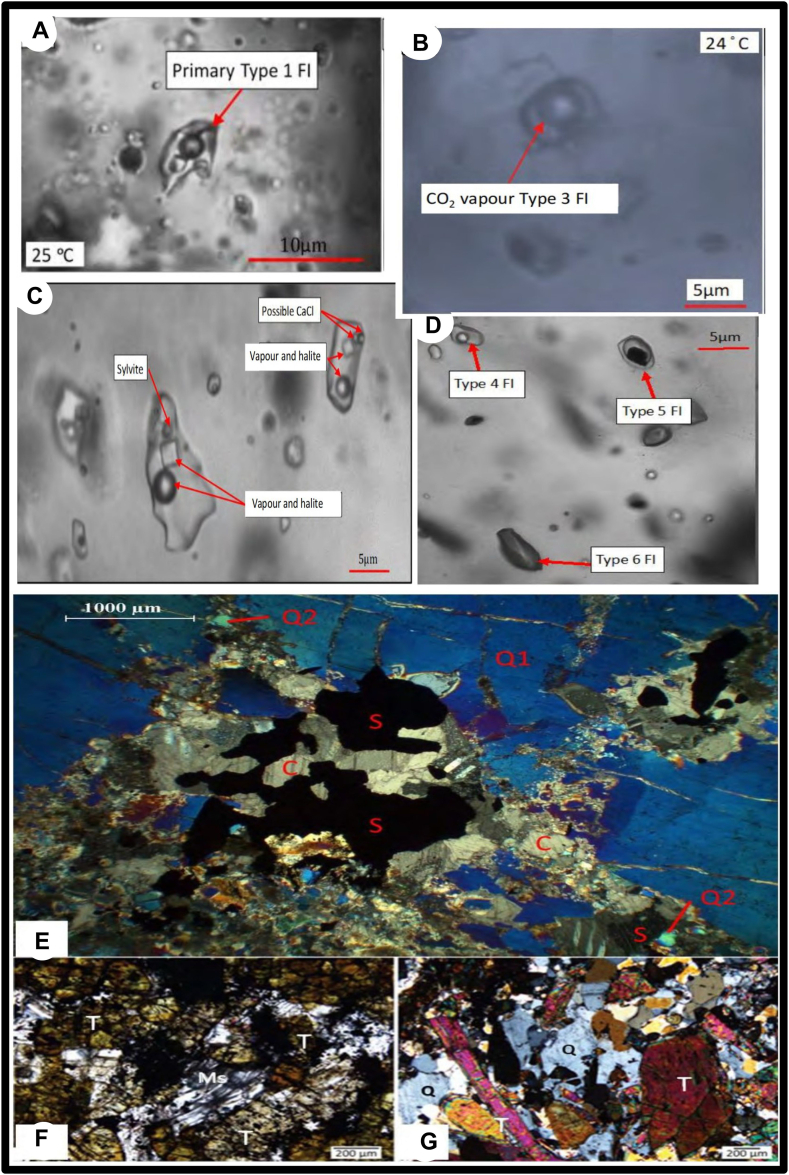
Representative images of major Fluid Inclusions types in TNGB and major mineral assemblages. A) Type 1 primary inclusion, B) Type 3 (CO_2_ vapor-rich bubble) primary inclusion, C) Type 2 (CO_2_-rich) primary inclusion associated with daughter crystals, D) Type 4,5 and 6 inclusions with some vapor-rich bubble, E) Primary quartz showing a tardy phase of sulfide and calcite and secondary quartz depicting an influx phase of sulfides and calcite, F) white mica crosscutting a no zoned tourmaline, G) Tourmaline-rich zone with recrystallized quartz (Q1 = Quartz primary; Q2 = Quartz secondary; T = Tourmaline, Ms = Muscovite, S= Sulfides, Calcite) (images credit: [34]).

During his investigation [34], found six types of fluids in Kamituga, Namoya, and Lugushwa deposits (Fig. 6(A-D)). These results are summarized in Table 1.

**Table 1:** Summary of the studied Fluid Inclusion in TNGB.

Fluid Inclusion type	*Salinity (wt%)*	Phases	T_ht_ (⁰C)	Rock unit	Location	Source
***Type 1:****(H*_*2*_*O-Nacl-CO*_*2*_*)*	26.3–50	L, V + halite daughter	390⁰C- 480 °C	Quartz-vein and tourmaline of both mineralized and no mineralized	Lugushwa	[34]
					Kamituga	
***Type 2:****(H*_*2*_*O–NaCl–CO*_*2*_	26.3–50	L, V, halite daughter	390⁰C-480 °C	Quartz veins close to tourmaline of both mineralized and no mineralized	Lugushwa	
					Kamituga	
***Type 3:****(H*_*2*_*O-Nacl-CO*_*2*_	<12	L (minor CO_2_), V_CO2_	390⁰C-480 °C	Quartz veins of both mineralized and unmineralized	Lugushwa	
					Namoya	
					Kamituga	
***Type 4:*** *H*_*2*_*O–CO*_*2*_	0–35	L, V	390⁰C-480 °C	Quartz veins of both mineralized and no mineralized	Kamituga,	
					Namoya,	
					Lugushwa	
***Type 5:*** *H*_*2*_*O-Nacl-CO*_*2*_	<10	L_CO2_, V_CO2_	390⁰C-480 °C	Quartz veins of both mineralized and no mineralized	Kamituga, Namoya and Lugushwa	
***Type 6:*** *H*_*2*_*O*	<18	L			Kamituga	
					Namoya	
					Lugushwa	
***Type 1:****(H*_*2*_*O-Nacl-CO*_*2*_	12.51 to 45.2	L, V_CO2_, L_CO2_, salt	375.2 °C to 386 °C	Quartz vein mineralized	Twangiza	[21]
***Type 2:****(H*_*2*_*O-Nacl-CO*_*2*_	12.51 to 45.2	L, V_CO2_, L_CO2_	375.2 °C to 386 °C	Mineralized quartz vein	Twangiza	
***Type 3:****(H*_*2*_*O-Nacl)*	5.56 to 11.1	L, L_CO2_, V, L, S.	279 °C to 386 °C	Quartz vein mineralized	Twangiza	
***Type 4:****(H*_*2*_*O-Nacl)*	5.56 to 11.1	L, V	279 °C to 386 °C	Quartz vein mineralized	Twangiza	
***Type 1:****(H*_*2*_*O–CO*_*2*_*)*		L, V_CO2_ rich Trace of CH4 and N2	370⁰C-400 °C	Pegmatites	Kamituga	[3]
***Type 2:****(H2O–CO2–CH4)*	Low salinity	L	380 °C	Diorite hosting quartz vein	Kamituga	
***Type 3:****(H2O-Nacl-CO2*	–	L, V, halite daughter crystals	–	Tourmaline quartz vein	Lugushwa	

Type 1 FIs are found in early-growing quartz -veins that include tourmaline, particularly in veins that are located in Lugushwa and Kamituga. Both mineralized and non-mineralized veins contain type 1. Its salinity ranges from 26.3 to 50 wt% Nacl equiv. and it is constraining 3 phases: the liquid-aqueous; aqueous-vapor bubble and halite phases. However, the vapor phase is never the dominating phase.

Similar to type 1, type 2 FIs are primarily found in unrecrystallized quartz. they are linked to early hydrothermal unrecrystallized quartz type 1. They are very close to the tourmaline. this type is found only in Kamituga and Lugushwa (Fig. 6(E-G)). Three phases are observed (liquid-aqueous, aqueous-vapor, solid halite daughter) and they are connected to both mineralized and unmineralized veins. they have a salinity ranging between 26.3 and 50 wt% Nacl equiv. Moreover, type 3 FIs are found in unmineralized and mineralized veins in hydrothermal and recrystallized quartz. their salinity is low (<12 wt% Nacl equiv.). three phases are noticed in these FIs type (Liquid-aqueous bearing some little trace of CO_2_); aqueous/CO_2_-vapor phases or liquid CO_2_ phases. Unlike types 1 and 2, they noticed the absence of halite daughter crystals in this type. It is noted that the highest value of gold mineralization clues was in agreement with type 3 FIs that contain the higher value of CO_2_ and lower value of saline level. CH_4_ elements were detectable in this type.

Type 3 FIs are observable throughout the entire TNGB. Type 4 FIs depicted two phases only (dominant aqueous liquid phases and aqueous-vapor phases). Its salinity ranges between 0 and 35 wt% Nacl.equiv.). They are detectable in both mineralized and no mineralized veins. like in type 3, gold higher content was noticed in samples with lower salinities and higher CO_2_. Furthermore, type 5 FIs showed three phases (aqueous liquid, a black solid (graphite, amorphous carbon, sulfides), and either CO_2_ liquid or an aqueous CO_2_-vapor phase). its salinity is lower (<10 wt% Nacl. equiv). They are observable in mineralized and no mineralized veins and are connected to the sulfide's phases (pyrite and arsenopyrite) in mineralized veins. Finally, the type 6 FIs possess only 1 phase (liquid phase) with the absence of a bubble visible. its salinity is < 18 wt% Nacl equiv. they are found in hydrothermal quartz veins together with type 3 and type 4. They are noticed in all the TNGB (Kamituga, Namoya, and Lugushwa).

Moreover [21], studied FIs in Twangiza gold fields. four types of FIs were constrained. The type 1 with H_2_O-Nacl-CO_2_. four phases were noticed (L-V_CO2_-L_CO2_ and Salt) inclusions characterized by carbonic phases (CO_2_ liquid, ± CO_2_ vapor). this type is considered a primary. The type 2 with H_2_O–NaCl–CO_2_ depicted three phases (L, V_CO2_, L_CO2_) or two-phase (L, V_CO2_) inclusions. it has an immiscible aqueous liquid CO_2_ and CO_2_ vapor. it is a primary inclusion. In addition, the type 3 has a H_2_O-Nacl. three phases (L, V, S) inclusions with liquid-rich aqueous solutions±CO_2_. Type 4 is a H_2_O-Nacl with two phases (L, V) inclusions with liquid-rich aqueous inclusions plus a CO_2_ vapor.

Furthermore [3], in order to understand the relationship between the gold mineralization in the TNGB as the gold was found to be at a proximal zone of sulfide and/or tourmaline veins, they systematically undertook a fluid inclusion study on tourmaline in Lugushwa and Kamituga. The study showed that primary pegmatites contain abundant FIs as compared to recrystallized quartz veins. Quartz of pegmatites is depicted to be CO_2_-rich fluid with L, V phases and some H_2_O. The homogenization temperature displayed to be around 370–400 °C. It is to be noted that CH_4_ and N_2_ were not noticed in this particular sample. In addition, a dioritic hosting a tourmalinic quartz vein showed CO_2_–H_2_O-rich fluids with CH_4_ dominating the liquid phase. The total homogenization of the analyzed sample happened around 380 °C. Saline abundantly with no halite or sylvite character was observable. In Lugushwa, the tourmaline quartz veins showed to be CO2-rich FIs with V and L phases. Halite crystals associated with these phases are saline brines.

### 3.2 Isotopic studies in the TNGB

[35] studied sulfur isotopes of the pyrites throughout the TNGB (Table 2). The results showed that, in the TNGB δ^34^ S range between – 18.4 ‰ and +22.6 ‰. The Twangiza gold fields showed an isotopic value of δ^34^ S in pyrite within host rock ranging between −2.2 ‰ and +3.0 ‰. In Kamituga gold fields, pyrite in host rocks depicted a value ranging between – 4.2 and – 0.6 ‰ while in Lugushwa gold fields a value of −18.4 ‰ to – 12.7 ‰. In Namoya pyrite in host rock displayed a value δ^34^ S of +12.4 ‰ to +22.6 ‰. Moreover, pyrite-bearing veins depicted a value of δ^34^S ranging between −5.2 ‰ and +3.0 ‰ in Twangiza gold fields. In Kamituga, a value of δ^34^ S ranging between −9.1 ‰ and – 7.4 ‰; δ^34^S ranging between −0.3 ‰ and +3.2 ‰ was noticed in Lugushwa gold fields. In Namoya, the δ^34^S value of +1.3 ‰ to +20.4 ‰.

**Table 2:** Summary of major Isotopes analyzed in TNGB.

Location	Host rock units (Metasedimentary, albitite, black shale)	Vein (Quartz veins)	Source
**δ**^**34**^**S Isotopes**			
Twangiza gold fields	−2.2 ‰ to +3.0 ‰ (n = 24)	−5.2 ‰ to +3.0 ‰ (n = 28)	[35]
Kamituga gold fields	−4.2 ‰ to −0.6 ‰ (n = 21)	−9.1 ‰ to −7.4 ‰ (n = 3)	
Lugushwa gold fields	−18.4 ‰ to −12.3 ‰ (n = 12)	−0.3 ‰ to +3.2 ‰ (n = 5)	
Namoya gold fields	+12.4 ‰ to +22.6 ‰ (n = 12)	+1.3 ‰ to +20.4 ‰ (n = 27)	
**δ**^**34**^**S Isotopes**			
Twangiza gold fields	1.03 ‰–8.50 ‰ (n = 10)	+2.62 ‰–3.37 ‰ (n = 8)	[21]
**δ**^**13**^**C isotope**			
Twangiza gold fields	−3.71 ‰ (Black shale)	−9.03 ‰ to −9.85 ‰ (n = 2)	[21]
**δ**^**11**^**B isotope**			
Lugushwa		−13.0 ‰ to −8.5 ‰ (Tourmaline vein)	[3]
Kamituga		−18.4 ‰ to −15.3 ‰ (Tourmaline vein)	

In Twangiza gold fields [21], studied the isotopic composition of pyrite within quartz-carbonate veins. The δ^34^ S of pyrites overall ranged between +1.03 ‰ and 8.48 ‰ with 95 % of the data being ranged from +2.62 ‰ to 3.37 ‰. Moreover, the δ^13^C on carbonate minerals showed a result of −3. 71 to – 9.85 ‰. Furthermore [3], with the objective of seeing if the hydrothermal tourmaline shared a common primary source similar to pegmatite, a boron isotopic study was conducted in Lugushwa gold fields and Kamituga. The δ^11^B of the tourmaline ranged between −18.4 ‰ and −15.3 ‰ in the Kamituga zone, whilst in Lugushwa a range of −13.0 ‰ to – 8.5 ‰ was noticed. Both gold fields showed a constant isotopic value according to the authors.

## 4 Genetic model

Given the results of the previous contributions, the TNGB is composed of the ore-forming fluid portrayed by low to high salinities (0–50 wt‰ Nacl equiv.) (Table 1). In addition, the ore-forming fluid seemed to be of medium temperature (270–400 °C). In Kamituga, Lugushwa, and Twangiza, type 1 and type 2 FIs portrayed a higher value in salinity (35–50 wt% Nacl equiv.) whilst type 3, 4,5,6 was lower in salinity (0–18 wt% Nacl equiv.).

According to Ref. [21], the higher salinity might be suspected of the ore fluid deriving from a magmatic source. He attributed the magmatic fluid origin to be linked to the underlain G4 granite of Kasika, which is convinced to be the parent rocks for the granite-pegmatites located in the line areas of Twangiza-Kamituga and Lugushwa. However, boiling might be the reason for the higher salinity observed in FIs of the TNGB, due to the coexistence of two phases within a single inclusion while they have different densities [40].

In TGNB, fluids are characterized mostly by H_2_O-Nacl-CO_2_ ± CH_4_ ± N_2._ Most of the liquid and vapor phases are dominated by CO_2_, and gold's higher grades seem to correlate with the high value of CO_2_ in FIs [34]. Therefore, the low salinity signatures and the petrographic composition of the FIs in TNGB are alike the orogenic gold-type deposits [41,42]. The circulation of these fluids enriched in CO_2_ together with H_2_O in channel pathways once in contact with host rocks triggered the sericitization and carbonatization alterations in the deposits [3,21,31]. Besides, the TNGB constitutes a wide range of geological formations, including granitic rocks, metapelites, and volcanic and volcano-sedimentary rocks, all of which possess the probability of producing gold during orogenic and collisional belt movements [[43], [44], [45]].

**Fig. 7:**
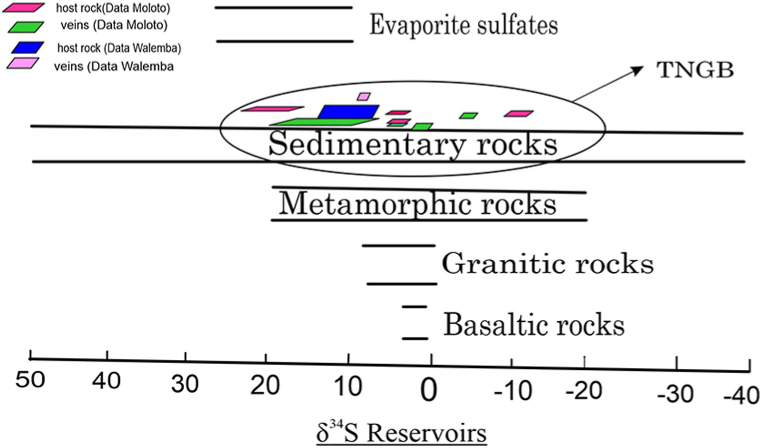
Sulfur Isotopes values of TNGB.

According to the isotopic results summarized in Table 2 and as portrayed in Fig. 7, the sulfur is likely to have the same source, which seems to be sedimentary [3,34]. It has been proved that the δ^34^S content of sulfide is constant during diagenesis and metamorphism in sedimentary terrains [46]. All the same, boron isotopic results from tourmaline as concluded by Ref. [3] were generated from sedimentary sources. This might be correlated with sulfur isotope outcomes as tourmaline has stability along the broad spectrum of pressure and temperatures, from the diagenetic process to the high-grade metamorphism of granulite facies [47].

There is no significant change between δ^34^S_bulk-pyrites_ in veins bearing ore minerals and the host rocks throughout the TNGB. This is consistent with the value of the isotopic bulk value of pyrites varying between −30 ‰ and −15 ‰ during the Proterozoic era [48]. However, some regions such as Namoya have been portrayed to be much heavier in pyrite Sulfur isotope both in host-rock and veins. An evaporite marine associated with a modern seawater environment might be suspicious to be the fact of that heavier isotopic signature [46,48]. Nevertheless, the δ^13^C in Twangiza gold fields depicted a value range that is consistence with the marine plus nonmarine organism signatures contributing to the ore-forming fluids [49].

[21] interpreted this phenomenon as the result of the combination of graphitic carbon with organic nature and carbonate minerals that were recycled in depths within the crust and taken up by magmatic ascending fluid. However, this statement created an ambiguity in understanding the role that played the magmatism inside the TNGB in gold ore-forming fluid and deposition. It is proven that most of the carbon in the crust occurs in the sedimentary rocks. A −5‰ value of δ^13^C is considered as an average in the crust. In addition, most of the diamonds found in eclogites have a δ^13^C value between −30 ‰ and - 5 ‰. A conclusion was established that the mantle is heterogeneous in carbon isotopes due to the reason that it can recycle during subduction [46].

An eclogite was detected in the Ubedian belt (Paleoproterozoic) in Tanzania with an age of 1.88-1.86 Ma, proof that an oceanic lithosphere subduction predated the continent-continent collision in Central Africa during the formation of Nuna supercontinent [30,50]. Therefore, the carbon isotopic observed in the Twangiza deposit is linked to a crustal nature rather than magmatic as quoted by Ref. [21]. All the mineralization ages reported in TNGB and Central-Africa metallogenic provinces showed that mineralization is linked Meso-Neoproterozoic model [8,19,30,50,51].

Geochronologically, an age of 900 Ma was determined for the early Neoproterozoic by using Rb–Sr geochronology on white mica of the cross-cutting post-folding quartz-vein in the Byumba gold deposit in Rwanda, northwestern Twangiza gold fields [52]. In Manono, Southward of KMP, Pegmatite (^40^Ar-^40^Ar muscovite) was dated 938.8 ± 5.1 Ma, and the endowment of Nb–Ta gave a U–Pb age of 940 ± 5.1 Ma [53]. In Kamituga, a pegmatite was dated 981 ± 16 Ma utilizing the Rb–Sr dating technique [3]. The albitite in Twangiza using Rb–Sr and Sm–Nd dating techniques gave an age of 1078 ± 27 Ma [21]. Nonetheless, compressional events are declared to occur in the entire KMP as consequences of the folding and thrusting of the metasediments [54].

Two main deformations are classified in the KMP linked to the mentioned compressional event. The D1 (1380 ± 15 Ma) and the D2 (1078 ± 9 Ma) compressional phases [1,2,55]. To sum up, the TNGB is categorized as a gold deposit of the orogenic type. A gold deposit hosted by sedimentary formations. The gold fields were compressed by tectonic activities resulting in folding, thrusting, and metamorphism of low greenschist facies that followed the pre-events of subduction and sedimentations.

**Fig. 8:**
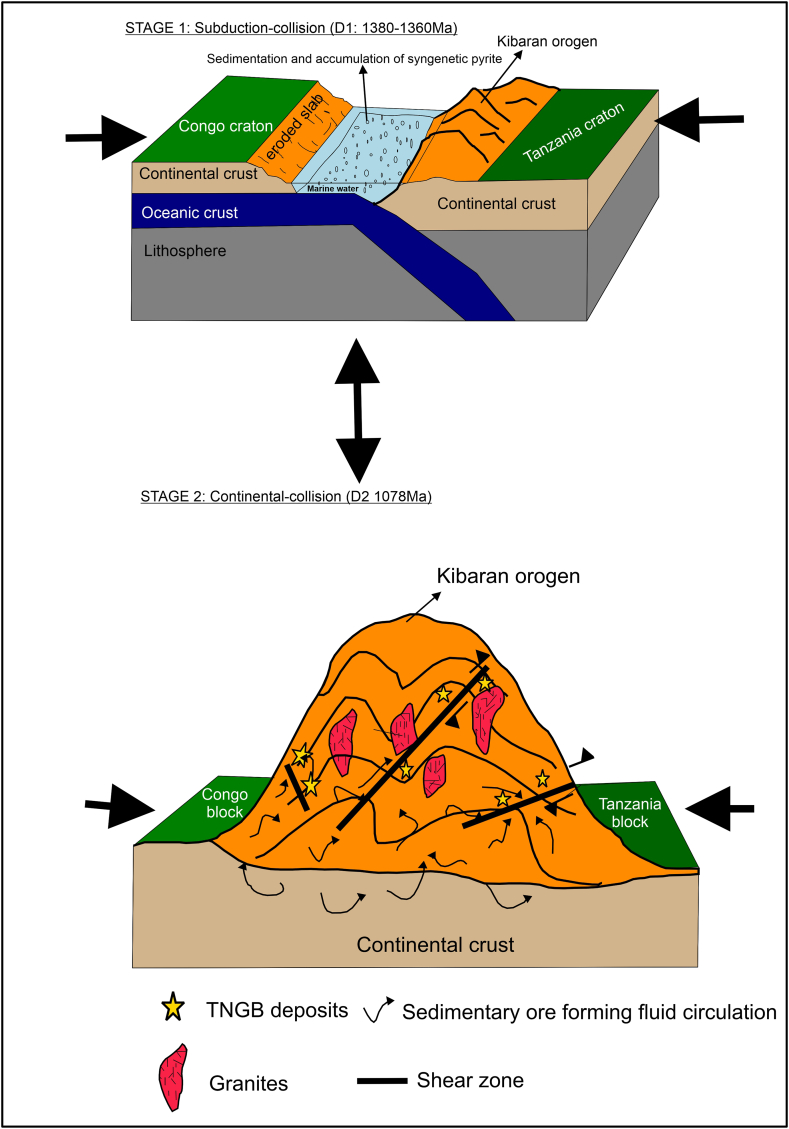
Genetic Sketch of TNGB gold deposits in Kibaran Metallogenic Province (DRC) (modified from Ref. [30]. (For interpretation of the references to colour in this figure legend, the reader is referred to the Web version of this article.)

Subduction and collision events in accretionary orogens are especially notable for the formation of gold deposits [42,56]. Following the lithology, structural controls, alteration zoning, and mineralogy of the gold fields occurring in TNGB, a carbonaceous sedimentary source-rock model for orogenic is worthy to be proposed as a suitable genetic model for the ore-forming process as proposed by Ref. [57]. Most of the analyzed samples used in previous contributions are carbonaceous metapelites [21,34,35]. [57] suggested an orogenic gold model of a two-stage basin scale. The TNGB, the gold is likely to have been formed in two stages (Fig. 8).

Stage 1 Sedimentation from material eroded from Congo craton: It has been assumed that sediments in Central Africa were deposited prior to the first metamorphism around 1.8 Ga, and were eroded from Tanzania and Congo craton during the Paleoproterozoic [3,50,58]. During the early epoch of accretion related to the Rodinia assembly around 1380-1360 Ma [8,30,59], Subduction took place due to the D1 compression event and at this period the continental basin margin was reduced and diagenesis continues in contribution under anoxic environment, pyrite is likely to get transform to pyrrhotite and gold might be shackle and concentrate through partitioning in sulfide-bearing minerals such as pyrite and arsenopyrite in marine bottom water into black shales and mudstones [57,60]. P_H_ is less than 6 in such conditions at t⁰ < 500 °C [46]. Therefore, in early diagenesis, H_2_S is the main sulfide complex to reacts with sediments to produce syngenetic and early pyrite [61,62].

Stage 2 Compression and medium-grade metamorphism of lower greenschist facies during the final stage of Rodinia assembly (D2 event around 1078). At this stage, the marine sea is closed, and the pyrite is transformed into pyrrhotite in carbonaceous shale. As the tectonic advanced, the gold retained in the lattice of sulfide-bearing minerals is released and dissolute into the hydrothermal waters that are flushed throughout the Kibaran orogen, and get deposited in structural sites of shear zones; breccias, zones proximal to black shales and fold zones. This is in agreement with the phase of enrichment of several gold deposits around the globe, where gold is accumulated in primary sediments and through multi-events of hydrothermal, and due to diffusion of gold, the carbonaceous sedimentary rocks are the main reservoir of sulfide-bearing metal enrichment [42,60,63].

## 5 Conclusions

In the current understanding, hosting in Kibaran Metallogenic Province (KMP) of the eastern Democratic Republic of Congo, Twangiza-Namoya Gold Belt (TNGB) is sourced from ore-forming fluids of medium temperature (350–500 °C), low salinity and from sedimentary fluids of H_2_O-Nacl-CO_2_ with a compound of CH_4_ and N_2_, confirming it genetic type as an orogenic gold deposit. The deposit has a style of emplacement similar to that of carbonaceous sedimentary hosted gold where the gold is syngenetic and long-lived with multiple stages of deposition. TNGB has two stages of deposition, the first stage is related to the D1 compressional event linked to the subduction during the early stage of Rodinia assembly. The later stage was the compressional event D2 related to continental collision during the final mechanism of the Rodinia assembly. TNGB has a mineralization age of a Neoproterozoic with a characteristic of vein-style and disseminated gold deposits.

## Ethical approval

This manuscript has not been published elsewhere in part or in entirety and is not under consideration by another journal. We have read and understood your journal's policies, and we believe that neither the manuscript nor the study violates any of these. We have not submitted our manuscript to a preprint server before submitting it to the Heliyon Journal.

## Consent to participate

Not applicable.

## Consent to publish

Not applicable.

## Data availability statement

The data used to infer new findings in this article are all portrayed in this article and are all referenced in.

## CRediT authorship contribution statement

**Rub'son N'nahano Heritier:** Writing – original draft. **Huan Li:** Writing – review & editing, Supervision. **Mohammed Abdalla Elsharif Ibrahim:** Writing – review & editing, Visualization, Software. **Claude Nambaje:** Writing – review & editing. **Moise Luemba:** Writing – review & editing.

## Declaration of competing interest

The authors declare that they have no known competing financial interests or personal relationships that could have appeared to influence the work reported in this paper.
